# The differences of the acromiohumeral interval between supine and upright radiographs of the shoulder

**DOI:** 10.1038/s41598-022-13632-0

**Published:** 2022-06-07

**Authors:** Prakasit Sanguanjit, Adinun Apivatgaroon, Phanuwat Boonsun, Surasak Srimongkolpitak, Bancha Chernchujit

**Affiliations:** Department of Orthopaedics, Faculty of Medicine, Thammasat University, Pathum Thani, Thailand

**Keywords:** Medical research, Epidemiology

## Abstract

The acromiohumeral interval (AHI) is a measurement used to determine the superior migration of the humeral head in rotator cuff (RC) tear patients. The purpose of this study was to compare the AHI of; supine, upright shoulder radiographs, and magnetic resonance imaging (MRI) of the shoulder. The 86 shoulders were divided into 3 groups that included; (1) non-full thickness tear (50%), (2) full thickness (FT) tear ≤3 cm (33.7%), and (3) FT tear > 3 cm (16.3%). The mean difference of AHI noted was significantly lower in the supine radiographs and MRIs than with the upright (1.34–1.37 mm, 1.62–1.87 mm, respectively). Upright AHI ≤ 7.0 mm had 27.9% sensitivity and 100% specificity in diagnosing FT tears with 64% accuracy (*p* < 0.001). The supine AHI ≤ 6.5 mm had 32.6% sensitivity, 100.0% specificity, and 66.3% accuracy (*p* < 0.01). The results revealed the AHI in supine radiographs were significantly lower than upright shoulder radiographs. For AHI ≤ 7 mm in upright shoulder radiographs, this remains as an appropriate diagnostic test for ruling in shoulders with full thickness rotator cuff tears. This value was not relevant for use as the cut point in the supine radiographs and MRIs.

## Introduction

Rotator cuff (RC) tears are one of the common shoulder problems seen in orthopaedic practice and while plain shoulder radiographs are the standard, initial investigation tools for rotator cuff tear patients, the acromion morphologies, os acromiale and the congruence of the glenohumeral articulation, are radiographic features used to evaluate the possibility of underlying pathologies. The superior migration of the humeral head (SMHH) is a phenomenon noted in late-stage rotator cuff tears. The pathology of an upward displacement of the humeral head is not clearly defined. Osseous structures and the capsulolabral complex are the main static stabilizers of the glenohumeral articulation while the rotator cuff, conjoint tendon and long head of the biceps play important roles in the dynamic glenohumeral stabilization^1^. The rotator cuff works in conjunction with deltoid muscle to create the force that couples around the glenohumeral joint^2^. Subscapularis (anterior) and infraspinatus/teres minor (posterior) create a balanced couple-force in the horizontal plane for centralizing the center of rotation of the humeral head during shoulder elevation. Increased deltoid pulls, lack of rotator cuff stabilization and the absence of the spacer by tendon structures may be the pathophysiologic causes of the SMHH^3,4^.

The acromiohumeral interval (AHI) is one of the measurement methods used to calculate the SMHH. The mean AHI in shoulders with an intact rotator cuff is approximately 10 mm (7–14 mm)^5,6^. While the AHI ≤ 7 mm measured on an anteroposterior radiograph suggests subacromial space narrowing and is indicative of large rotator cuff tears. In these cases, the likelihood of successful outcomes after the repair are reduced^4–6^. A seated or standing AHI less than 6 mm is indicative of a longstanding, total infraspinatus tear^7^.

The AHI value may be influenced by gravity during upright arm position and this has the possibility of obtaining false negative results in detecting rotator cuff tears. Measurement of the AHI from supine MRIs is lower than measurements seen in upright shoulder radiographs^8^ but plain radiographs are still the best initial investigation for rotator cuff-related patients. Supine shoulder radiographs may have less of a chance of confounding the AHI measurement by gravity and may reduce false negative detection of rotator cuff tears.

The purpose of this study was to compare the AHI of supine shoulder radiographs, upright shoulder radiographs, and magnetic resonance imaging (MRI) of shoulders with the MRI findings of the rotator cuff pathologies/tears. The sensitivity, specificity, and accuracy of the measured AHI in detecting full thickness RC tears by MRI were evaluated.

## Materials and methods

The study population consisted of 86 patients (34 men, 52 women; mean age, 61.06 years). All shoulder radiographs and MRIs were performed at Thammasat University Hospital, from the period of July 2020 to May 2021, were compared. The inclusion criteria were patients more than 18 years of age and patients with suspected rotator cuff related shoulder pain who underwent an MRI of the affected shoulder. The exclusion criteria were patients who had a fracture around the shoulder, a history of shoulder surgery, severe osteoarthritis, rotator cuff tear arthropathy, and/or a history of septic or inflammatory arthritis in the shoulder.

### Imaging protocol

*Shoulder radiograph *(*Grashey view*): Radiographs were performed with a Simens X-ray vacuum technology (Simens Healthcare, Jiangsu China). Assessment included true anteroposterior radiographs with the arm in a neutral position. The patients’ scapula parallel to the image receptor (rotating the body 35°- 45° forward). The beam was angled 20° craniocaudally. All radiographs were prospectively acquired with the patient in the upright and supine positions.

*MRI shoulder:* The MRI examinations of the shoulders were performed in a supine position with a MAGNETOM Skyra is 3 T MRI, Siemens Healthcare Headquarters (Siemens Healthcare GmbH Henkestr, Erlangen Germany). The same units and the same protocols were used on all shoulder MRIs during the study period. The imaging planes were as follows; transverse, coronal oblique (perpendicular to the glenoid) and sagittal oblique (parallel to the glenoid). The Picture Archiving and Communication System (PACs), Synapse program (Fujifilm Medical Systems Inc., Hanover Park, Illinois) were used to make all measurements, on both radiographs and MRIs.

### Measurement of acromiohumeral interval

Two blinded orthopaedic surgeons, separately measured the AHI on shoulder radiographs (Grashey view) and on the sagittal oblique MRI. The process of reviewing the images required the Picture Archiving and Communication System (PACS) work station. The AHI was measured in millimeters. The interpretations by both reviewers consisted of the assessment of inter-observer and intra-observer reliability, the interval period of measurement was 2 weeks. The AHI of the shoulder radiographs was measured by using the distance between the dense cortical bone at the inferior aspect of the acromion and the subchondral lamina of the humeral head^3^. The shortest distance was measured. The same measurement was performed by reviewers on sagittal oblique T1-weighted MRI. The AHI was measured at the shortest distance between the lowest part of the acromion and the center of the subchondral cortex of the humeral head^8^.

### Patient grouping

The gold standard for diagnoses of rotator cuff pathologies/tears is an MRI of the affected shoulder. Patients were classified in 3 groups as measured by a fellowship-trained, sports medicine surgeon, in accordance to the antero-posterior tear size of posterosuperior rotator cuff in the sagittal MRI shoulder. Group 1; non-full thickness tear including RC tendinosis, subacromial bursitis, partial thickness RC tear. Group 2; full thickness tear ≤ 3 cm. Group 3; full thickness tear > 3 cm.

### Statistical analysis

Statistical analysis was performed using SPSS (Version 25, IBM, Armonk, New York, USA) Demographic data, quantitative data (age, height, weight, BMI and size of rotator cuff tear) are represented by mean ± standard deviation (SD). Qualitative or categorical data (sex, site and number of patients in each group) represented in percentages.

Comparison of the AHI in radiographs and MRI: *p-value* by ANOVA test and Paired t-test were used for assessing differences in the AHI between shoulder radiographs in the supine and upright positions, and MRIs of the shoulder. A *p* value < 0.05 was considered statistically significant.

The inter- and intra-rater reliability of the AHI measurement in 3 views were evaluated using the intraclass correlation coefficient (ICC). The ICC values < 0.5, 0.5–0.75, 0.75–0.9, > 0.90 are indicative of poor, moderate, good, and excellent reliability, respectively^9^.

The sensitivity and specificity of the AHI measurement in rotator cuff tears were calculated the ROC values and presented as an area under the curve (AUC) of the AHI measurement from upright radiographs and supine shoulder radiographs.

### Compliance with ethical standards

All procedures performed in studies involving human participants were in accordance with the ethical standards of the ethics committee of the Thammasat University Hospital in accordance with relevant guidelines and regulations. The study was approved by the ethics committee of the Thammasat University Hospital (Registration no. MTU-EC-OT-2-031/64). Informed consent was obtained from all subjects.

## Results

Eighty-six consecutive radiographs and MRI imaging sets were recorded between July, 2020 and May, 2021 and were evaluated according to the inclusion and exclusion criteria. The mean age was 61.1 ± 11.4 years. Mean BMI was 24.54. There were 52 (60.5%) women. There were 58.1% of the patients right site affected. The mean tear size was 1.28 cm with median of 0.7 cm (range 0–5.4 cm).

The 86 shoulders were divided to 3 groups. The predominance of the study population was with non-full thickness rotator cuff tears (NFT) (50%), while 33.7% were full thickness tears (FT) ≤ 3 cm, and 16.3% were full thickness tears > 3 cm (Table 1).

**Table 1:** Summary of the demographic data of patients.

Variables	Statistics
Age	61.1 ± 11.4
Height (cm)	161.8 ± 7.1
Weight (kg)	64.4 ± 10.7
BMI	24.54 ± 3.48
**Sex**	
Male	34 (39.5%)
Female	52 (60.5%)
**Side**	
Right	50 (58.1%)
Left	36 (41.9%)
**Tear size (cm)**	1.28 ± 1.46
Median (range)	0.7 (0–5.4)
**Group**	
Non-full thickness tear	43 (50%)
Full thickness tear $\le \hspace{0.17em}$3 cm	29 (33.7%)
Full thickness tear > 3 cm	14 (16.3%)

According to their patient groupings, the patients in the full thickness tear groups were older than those in the non-full thickness tear group (*p* = 0.011). Gender and BMI showed no significant differences between groups. Right shoulders were affected in full-thickness tear patients more frequently than in those with non-full thickness tears. There were significant differences in tear and retraction sizes for each group (*p* < 0.001) (Table 2).

**Table 2:** Summarizes the demographic data according to tear size group. (NFT = non-full thickness tear, FT = full thickness tear).

	NFT	FT $\le \hspace{0.17em}$3 cm	FT > 3 cm	*p* value
N	43	29	14	
Tear size (cm)	0	1.31 ± 0.47	4.21 ± 0.82	< 0.001
Retraction size (cm)	N/A	1.72 ± 0.94	3.64 ± 0.68	< 0.001
Age	57.6 ± 12.2	65.6 ± 10.1	62.3 ± 7.8	0.011
BMI	24.88 ± 3.07	24.23 ± 3.28	24.13 ± 5	0.666
**Sex**				
Male	13 (30.2%)	16 (55.2%)	5 (35.7%)	0.100
Female	30 (69.8%)	13 (44.8%)	9 (64.3%)	
**Side**				
Right	16 (37.2%)	22 (75.9%)	12 (85.7%)	< 0.001
Left	27 (62.8%)	7 (24.1%)	2 (14.3%)	

*p* value obtained by ANOVA test and Chi-square test.

### AHI measurement

Overall, there was a significant difference *(p* < 0.001) in the AHI measurements according to tear size groups in supine & upright position shoulder radiographs (Grashey view) and shoulder MRIs.

### Comparison of AHI: (Table 3)

**Table 3:** Summarizes the AHI measurement according to tear size group. (NFT = non-full thickness tear, FT = full thickness tear).

	NFT	FT $\le \hspace{0.17em}$3 cm	FT > 3 cm	*p* value
N	43	29	14	
AHI (upright) (mm)	10.09 ± 1.67	9.38 ± 1.87	8.1 ± 3.02	0.006
AHI (supine) (mm)	8.73 ± 1.45	8.03 ± 1.48	6.75 ± 2.69	0.001
AHI (MRI) (mm)	8.48 ± 1.43	7.7 ± 1.41	6.23 ± 2.37	< 0.001
**Upright versus supine**				
Mean difference (95% CI) (mm)	1.37 (1.09, 1.64)	1.35 (1.08, 1.61)	1.34 (0.87, 1.82)	
*p* value	< 0.001	< 0.001	< 0.001	
**Upright versus MRI**				
Mean difference (95% CI) (mm)	1.62 (1.2, 2.03)	1.68 (1.14, 2.23)	1.87 (0.86, 2.88)	
*p* value	< 0.001	< 0.001	0.002	
**Supine versus MRI**				
Mean difference (95% CI) (mm)	0.25 (− 0.05, 0.55)	0.34 (− 0.06, 0.73)	0.53 (− 0.27, 1.33)	
*p* value	0.099	0.091	0.177	

*p* value by ANOVA test and Paired t test.

There was a significant difference of the AHI obtained from the upright and supine shoulder radiographs in all groups. The mean differences were lower in the supine radiographs (1.34–1.37 mm).

There was a significant difference of the AHI obtained from the upright shoulder radiographs and MRIs in both groups. The mean differences were lower in the MRIs (1.62–1.87 mm).

There were no significant differences of the AHI obtained from the supine radiographs and MRIs (0.25–0.53 mm). Our findings indicate that AHI measurement from supine shoulder radiographs was equivalent in predictive value to the MRI measurement and lower than from upright shoulder radiographs.

**Figure 1:**
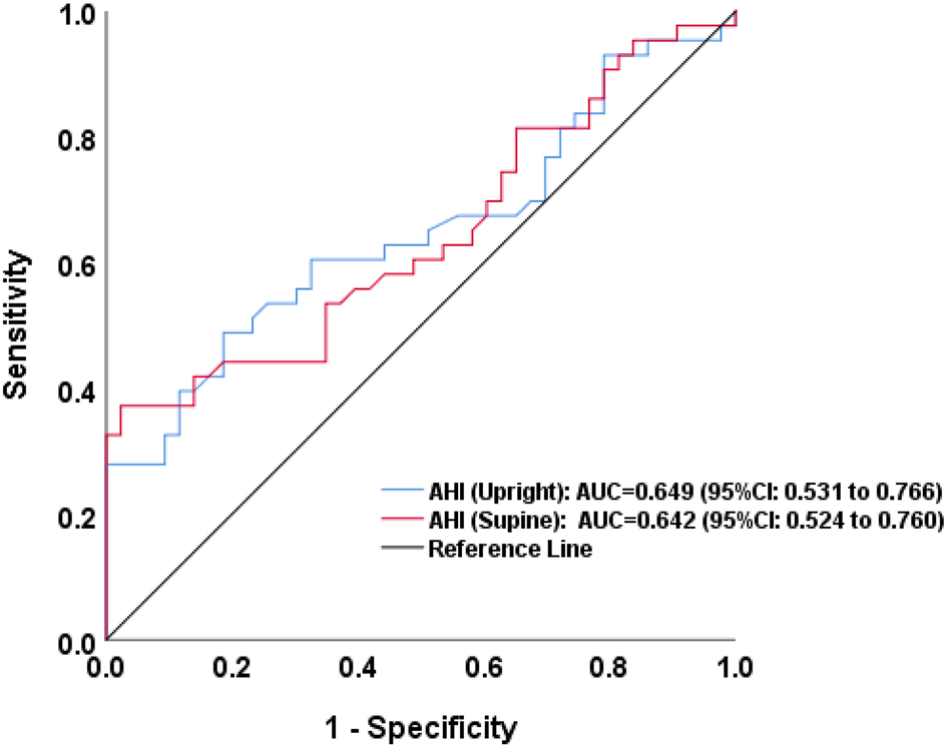
ROC curve of the AHI measurement from upright and supine radiographs.

When studying sensitivity and specificity of the AHI measurement in rotator cuff tears using ROC analysis, the result showed an area under the curve (AUC) of 0.649 (95% confidence interval 0.53–0.77) in the AHI measurement from upright radiographs and an AUC of 0.642 (95% confidence interval 0.52–0.76) from supine radiographs (Fig. 1). The AUC demonstrated the poor overall test accuracy of the AHI in determination of RC tears.

Analysis of the cut off value of the AHI in diagnosis of a full thickness rotator cuff tear from upright and supine radiographs are calculated (Appendix 1). With the AHI value ≤ 7.09 mm in upright radiographs, the sensitivity and specificity to diagnose full thickness superoposterior rotator cuff tears were 27.9% and 100.0%, respectively, with 64% accuracy (*p* < 0.001). The AHI cut off value of ≤ 9.52 mm in upright radiographs had 60.5% sensitivity, 67.4% specificity, and 64% accuracy (*p* = 0.01). In the supine radiographs, the AHI ≤ 6.56 mm had 32.6% sensitivity, 100.0% specificity, and 66.3% accuracy (*p* < 0.01). Using an AHI cut off value of ≤ 7.42 mm, the sensitivity and specificity in diagnosing full thickness superoposterior rotator cuff tears were 41.9% and 86.0%, respectively, with 64.0% accuracy (*p* = 0.004).

Regarding interrater reliability and validity of the measurements^9^, the ICC of the individual measures between the two examiners, the inter- and intra-rater reliability of the AHI measurement in 3 views have shown “moderate to good reliability” (0.668–0.824) (Table 4).

**Table 4:** Summarizes the inter/ intra observer reliability, AHI = acromiohumeral interval, CI = confidence interval.

	ICC	95% CI
**Inter reliability**		
AHI(upright)	0.824	0.739–0.878
AHI (supine)	0.774	0.689–0.834
AHI (MRI)	0.747	0.642–0.82
**Intra reliability**		
AHI(upright)	0.753	0.666–0.817
AHI (supine)	0.668	0.551–0.754
AHI (MRI)	0.697	0.591–0.776

## Discussion

This study has also found that the AHI measurements, in either supine or upright radiographs and MRIs, were reliable and reproducible. The AHI is the shortest distance between the inferior cortex of the acromion and the top of the humeral head, the normal AHI is 7–14 mm^5,6^. Patients with an AHI ≤ 7 mm suggests a large rotator cuff tear^4,6^ and < 6 mm suggests a longstanding, total infraspinatus tear^7^. Gruber et al. measured AHI on standard AP radiographs and found that the AHI measurement was reliable and reproducible^10^. The AHI measurement from standing or seated X-ray views that representing the upright position of the shoulders shows satisfactory reproducibility^11^.

From previous studies, Oliveira et al. evaluated the AHI in shoulder MRIs and found that the AHI on MRIs is not influenced by gravity, degree of superior migration in relation to size, retraction and topography of the rotator cuff tear^3^. Merzayan et al. found significant differences in AHI measurements between radiographs and MRIs of the same shoulder with a massive rotator cuff tear. The AHI was lower on MRIs when compared with radiographs^8^. As gravity may affect to the value of AHI and the weight of arm may lead to false negative results with some specific AHI cut-off values (such as the presenting of FT RC tear while upright AHI of > 7 mm).

In this study, the AHI measurement in supine radiographs and MRIs were less than the AHI measured in upright shoulder radiographs due to the effect of gravity on the arm. An AHI value ≤ 7 mm in upright radiographs did not have the same cut point value as in the supine radiographs and MRIs in the diagnosis of FT rotator cuff tears. The AHI measurement of upright shoulder radiographs was higher compared with the supine films and the MRI with a significant difference (*p* < 0.001)*.* There was no difference in the comparison of the AHI from supine shoulder radiographs and MRIs. This finding is in agreement with previous studies^3,12^.

The AHI measurement from upright, supine radiographs, and MRIs correlates with the rotator cuff tear size. The AHI had a lower value in larger tears than in the smaller tears. Similar to the study comparing AHI values of plain radiographs and MRIs on bilateral shoulders of unilateral RC tears^13^, it has been shown the mean AHI was significant narrowing in the shoulder with RC tear. The mean AHI on radiographs were 6.93 mm and 9.11 mm, on MRIs were 5.94 mm and 7.46 mm in the patient and control groups, respectively. The AHI value was significantly reduced by increasing the severity of supraspinatus tendon retraction.

Regarding the AUC of our study, the AHI measurement from upright, supine radiographs, and MRIs had poor overall accuracy. So, AHI measurement, with any other radiographic methods, would not be suitable in the single standard method for the use of detecting a superoposterior rotator cuff tear. Other than in the RC tear, the AHI value may decrease in the adhesive capsulitis of the shoulders (9.3 ± 1.3 mm) when compared to the controls (11.0 ± 1.7 mm) (*p* < 0.001)^14^.

In the upright AHI, the low sensitivity (27.9%) of the AHI ≤ 7 mm in diagnosing an FT rotator cuff tear represents the limitations for the value of the upright AHI to be used as a screening tool for detecting a FT rotator cuff tear. While the 100% specificity represents a valuable tool as diagnostic test for ruling in the FT RC tear^15^, this statistical analysis was based on a 64% accuracy. The AHI cut off value of ≤ 9.52 mm had 60.5% sensitivity, 67.4% specificity, and 64% accuracy (*p* = 0.01).

Compared with previous publications, an AHI measurement less than 7 mm was considered abnormal in several publications^16^. Goupille et al.^17^ reported that an AHI of 7 mm or less on standard shoulder radiographs had a specificity of 98% with a low sensitivity of 24% for RC tear. Goutallier et al.^7^ showed that an AHI of < 6 mm had high specificity, but very low sensitivity and therefore no diagnostic value for AHI as a screening tool in the indication of RC tears. The retrospective MRI analysis showed the mean AHI in the impingement group was significantly lower than control group (6.8 mm ± 1.0 mm vs. 10.1 mm ± 1.5 mm, *p* < 0.001) and revealed a positive and moderate correlation between subacromial volume and AHI (R = 0.6; *p* = 0.01)^18^.

In the supine AHI, the measurement value was lower than in the upright radiographs. The AHI ≤ 6.56 mm had 100% specificity while 32.6% sensitivity, and 66.3% accuracy (*p* < 0.01) for ruling in a FT RC tear. Using an AHI cut off value of ≤ 7.42 mm, the sensitivity and specificity for diagnosing a FT, superoposterior rotator cuff tear were 41.9% and 86.0% respectively with 64.0% accuracy (*p* = 0.004). Although, the supine AHI had equivalency in predictive value to the MRI measurement and was lower than that obtained from upright shoulder radiographs. However, the AHI measurement from the supine shoulder radiographs may not have any additional value in the diagnosis of rotator cuff tears when compared to common standard upright shoulder radiographs.

The strength of this study is that this is the first study to analyze the effects of gravity in plain shoulder radiographs with the use of AHI measurement in correlation to the presentation of a FT, superoposterior rotator cuff tear. This study has some limitations, the number of patients in each tear size group is different, the non-full thickness tear group was younger in age generally than the full thickness tear group, and there were smaller numbers of patients with large to massive tear sizes (FT > 3 cm). These may have an effect on the interpretation of outcomes.

## Conclusion

The AHI seen in supine radiographs were significantly lower than noted in the upright shoulder radiographs. For AHI ≤ 7 mm, upright shoulder radiographs remain an appropriate diagnostic test for ruling in full thickness rotator cuff tears. However, this value was not relevant for use as the cut point in the supine radiographs and MRIs of the shoulders.

## Supplementary Information

Supplementary Information.

## Data Availability

The authors confirm that the data supporting the findings of this study are available within the article and its supplementary material.
