# Supplementary material for: The differences of the acromiohumeral interval between supine and upright radiographs of the shoulder
Source: Sci Rep. 2022 Jun 7;12:9404. doi: 10.1038/s41598-022-13632-0 (PMC9174172; doi:10.1038/s41598-022-13632-0)
Supplement: Supplementary file 1 — Supplementary Information. [file 41598_2022_13632_MOESM1_ESM.docx]

| **Variable(s)** | **cut off**  $\leq$ | **TP** | **FP** | **FN** | **TN** | **Sensitivity** | **Specificity** | **PPV** | **NPV** | **Accuracy** | **p-value** |
| --- | --- | --- | --- | --- | --- | --- | --- | --- | --- | --- | --- |
| AHI (Upright) | 14.70 | 42 | 43 | 1 | 0 | 97.7% | 0.0% | 49.4% | 0.0% | 48.8% | 0.315 |
|  | 14.14 | 42 | 42 | 1 | 1 | 97.7% | 2.3% | 50.0% | 50.0% | 50.0% | 1 |
|  | 13.71 | 41 | 42 | 2 | 1 | 95.3% | 2.3% | 49.4% | 33.3% | 48.8% | 0.557 |
|  | 13.20 | 41 | 41 | 2 | 2 | 95.3% | 4.7% | 50.0% | 50.0% | 50.0% | 1 |
|  | 12.92 | 41 | 40 | 2 | 3 | 95.3% | 7.0% | 50.6% | 60.0% | 51.2% | 0.645 |
|  | 12.79 | 41 | 39 | 2 | 4 | 95.3% | 9.3% | 51.3% | 66.7% | 52.3% | 0.397 |
|  | 12.68 | 41 | 38 | 2 | 5 | 95.3% | 11.6% | 51.9% | 71.4% | 53.5% | 0.237 |
|  | 12.60 | 41 | 37 | 2 | 6 | 95.3% | 14.0% | 52.6% | 75.0% | 54.7% | 0.138 |
|  | 12.17 | 40 | 37 | 3 | 6 | 93.0% | 14.0% | 51.9% | 66.7% | 53.5% | 0.291 |
|  | 11.71 | 40 | 36 | 3 | 7 | 93.0% | 16.3% | 52.6% | 70.0% | 54.7% | 0.178 |
|  | 11.55 | 40 | 35 | 3 | 8 | 93.0% | 18.6% | 53.3% | 72.7% | 55.8% | 0.107 |
|  | 11.47 | 40 | 34 | 3 | 9 | 93.0% | 20.9% | 54.1% | 75.0% | 57.0% | 0.062 |
|  | 11.45 | 38 | 34 | 5 | 9 | 88.4% | 20.9% | 52.8% | 64.3% | 54.7% | 0.243 |
|  | 11.29 | 37 | 34 | 6 | 9 | 86.0% | 20.9% | 52.1% | 60.0% | 53.5% | 0.394 |
|  | 11.05 | 36 | 34 | 7 | 9 | 83.7% | 20.9% | 51.4% | 56.3% | 52.3% | 0.579 |
|  | 10.96 | 36 | 33 | 7 | 10 | 83.7% | 23.3% | 52.2% | 58.8% | 53.5% | 0.417 |
|  | 10.90 | 36 | 32 | 7 | 11 | 83.7% | 25.6% | 52.9% | 61.1% | 54.7% | 0.289 |
|  | 10.84 | 35 | 32 | 8 | 11 | 81.4% | 25.6% | 52.2% | 57.9% | 53.5% | 0.436 |
|  | 10.77 | 35 | 31 | 8 | 12 | 81.4% | 27.9% | 53.0% | 60.0% | 54.7% | 0.307 |
|  | 10.69 | 34 | 31 | 9 | 12 | 79.1% | 27.9% | 52.3% | 57.1% | 53.5% | 0.451 |
|  | 10.66 | 33 | 31 | 10 | 12 | 76.7% | 27.9% | 51.6% | 54.5% | 52.3% | 0.621 |
|  | 10.63 | 33 | 30 | 10 | 13 | 76.7% | 30.2% | 52.4% | 56.5% | 53.5% | 0.465 |
|  | 10.59 | 32 | 30 | 11 | 13 | 74.4% | 30.2% | 51.6% | 54.2% | 52.3% | 0.631 |
|  | 10.54 | 31 | 30 | 12 | 13 | 72.1% | 30.2% | 50.8% | 52.0% | 51.2% | 0.812 |
|  | 10.47 | 30 | 30 | 13 | 13 | 69.8% | 30.2% | 50.0% | 50.0% | 50.0% | 1 |
|  | 10.37 | 30 | 29 | 13 | 14 | 69.8% | 32.6% | 50.8% | 51.9% | 51.2% | 0.816 |
|  | 10.32 | 29 | 28 | 14 | 15 | 67.4% | 34.9% | 50.9% | 51.7% | 51.2% | 0.820 |
|  | 10.26 | 29 | 27 | 14 | 16 | 67.4% | 37.2% | 51.8% | 53.3% | 52.3% | 0.651 |
|  | 10.20 | 29 | 26 | 14 | 17 | 67.4% | 39.5% | 52.7% | 54.8% | 53.5% | 0.501 |
|  | 10.12 | 29 | 25 | 14 | 18 | 67.4% | 41.9% | 53.7% | 56.3% | 54.7% | 0.372 |
|  | 10.05 | 29 | 24 | 14 | 19 | 67.4% | 44.2% | 54.7% | 57.6% | 55.8% | 0.268 |
|  | 9.99 | 28 | 22 | 15 | 21 | 65.1% | 48.8% | 56.0% | 58.3% | 57.0% | 0.190 |
|  | 9.91 | 27 | 22 | 16 | 21 | 62.8% | 48.8% | 55.1% | 56.8% | 55.8% | 0.276 |
|  | 9.88 | 27 | 21 | 16 | 22 | 62.8% | 51.2% | 56.3% | 57.9% | 57.0% | 0.193 |
|  | 9.85 | 27 | 20 | 16 | 23 | 62.8% | 53.5% | 57.4% | 59.0% | 58.1% | 0.130 |
|  | 9.79 | 27 | 19 | 16 | 24 | 62.8% | 55.8% | 58.7% | 60.0% | 59.3% | 0.084 |
|  | 9.73 | 26 | 19 | 17 | 24 | 60.5% | 55.8% | 57.8% | 58.5% | 58.1% | 0.131 |
|  | 9.69 | 26 | 18 | 17 | 25 | 60.5% | 58.1% | 59.1% | 59.5% | 59.3% | 0.084 |
|  | 9.62 | 26 | 16 | 17 | 27 | 60.5% | 62.8% | 61.9% | 61.4% | 61.6% | 0.031 |
|  | 9.52 | 26 | 14 | 17 | 29 | 60.5% | 67.4% | 65.0% | 63.0% | 64.0% | 0.010 |
|  | 9.46 | 25 | 14 | 18 | 29 | 58.1% | 67.4% | 64.1% | 61.7% | 62.8% | 0.017 |
|  | 9.43 | 24 | 14 | 19 | 29 | 55.8% | 67.4% | 63.2% | 60.4% | 61.6% | 0.030 |
|  | 9.37 | 24 | 13 | 19 | 30 | 55.8% | 69.8% | 64.9% | 61.2% | 62.8% | 0.017 |
|  | 9.25 | 23 | 13 | 20 | 30 | 53.5% | 69.8% | 63.9% | 60.0% | 61.6% | 0.029 |
|  | 9.17 | 23 | 12 | 20 | 31 | 53.5% | 72.1% | 65.7% | 60.8% | 62.8% | 0.016 |
|  | 9.16 | 23 | 11 | 20 | 32 | 53.5% | 74.4% | 67.6% | 61.5% | 64.0% | 0.008 |
|  | 9.12 | 22 | 10 | 21 | 33 | 51.2% | 76.7% | 68.8% | 61.1% | 64.0% | 0.007 |
|  | 8.94 | 21 | 10 | 22 | 33 | 48.8% | 76.7% | 67.7% | 60.0% | 62.8% | 0.014 |
|  | 8.79 | 21 | 9 | 22 | 34 | 48.8% | 79.1% | 70.0% | 60.7% | 64.0% | 0.007 |
|  | 8.76 | 21 | 8 | 22 | 35 | 48.8% | 81.4% | 72.4% | 61.4% | 65.1% | 0.003 |
|  | 8.73 | 20 | 8 | 23 | 35 | 46.5% | 81.4% | 71.4% | 60.3% | 64.0% | 0.006 |
|  | 8.67 | 19 | 8 | 24 | 35 | 44.2% | 81.4% | 70.4% | 59.3% | 62.8% | 0.011 |
|  | 8.62 | 18 | 8 | 25 | 35 | 41.9% | 81.4% | 69.2% | 58.3% | 61.6% | 0.019 |
|  | 8.53 | 18 | 7 | 25 | 36 | 41.9% | 83.7% | 72.0% | 59.0% | 62.8% | 0.009 |
|  | 8.41 | 17 | 6 | 26 | 37 | 39.5% | 86.0% | 73.9% | 58.7% | 62.8% | 0.007 |
|  | 8.37 | 17 | 5 | 26 | 38 | 39.5% | 88.4% | 77.3% | 59.4% | 64.0% | 0.003 |
|  | 8.34 | 16 | 5 | 27 | 38 | 37.2% | 88.4% | 76.2% | 58.5% | 62.8% | 0.006 |
|  | 8.11 | 14 | 5 | 29 | 38 | 32.6% | 88.4% | 73.7% | 56.7% | 60.5% | 0.019 |
|  | 7.87 | 14 | 4 | 29 | 39 | 32.6% | 90.7% | 77.8% | 57.4% | 61.6% | 0.008 |
|  | 7.80 | 13 | 4 | 30 | 39 | 30.2% | 90.7% | 76.5% | 56.5% | 60.5% | 0.015 |
|  | 7.64 | 12 | 4 | 31 | 39 | 27.9% | 90.7% | 75.0% | 55.7% | 59.3% | 0.027 |
|  | 7.51 | 12 | 3 | 31 | 40 | 27.9% | 93.0% | 80.0% | 56.3% | 60.5% | 0.011 |
|  | 7.45 | 12 | 2 | 31 | 41 | 27.9% | 95.3% | 85.7% | 56.9% | 61.6% | 0.004 |
|  | 7.33 | 12 | 1 | 31 | 42 | 27.9% | 97.7% | 92.3% | 57.5% | 62.8% | 0.001 |
|  | 7.09 | 12 | 0 | 31 | 43 | 27.9% | 100.0% | 100.0% | 58.1% | 64.0% | <0.001 |
|  | 6.93 | 11 | 0 | 32 | 43 | 25.6% | 100.0% | 100.0% | 57.3% | 62.8% | <0.001 |
|  | 6.80 | 10 | 0 | 33 | 43 | 23.3% | 100.0% | 100.0% | 56.6% | 61.6% | 0.001 |
|  | 6.63 | 9 | 0 | 34 | 43 | 20.9% | 100.0% | 100.0% | 55.8% | 60.5% | 0.002 |
|  | 6.45 | 8 | 0 | 35 | 43 | 18.6% | 100.0% | 100.0% | 55.1% | 59.3% | 0.003 |
|  | 6.29 | 7 | 0 | 36 | 43 | 16.3% | 100.0% | 100.0% | 54.4% | 58.1% | 0.006 |
|  | 6.00 | 6 | 0 | 37 | 43 | 14.0% | 100.0% | 100.0% | 53.8% | 57.0% | 0.011 |
|  | 5.71 | 5 | 0 | 38 | 43 | 11.6% | 100.0% | 100.0% | 53.1% | 55.8% | 0.021 |
|  | 5.65 | 4 | 0 | 39 | 43 | 9.3% | 100.0% | 100.0% | 52.4% | 54.7% | 0.041 |
|  | 5.60 | 3 | 0 | 40 | 43 | 7.0% | 100.0% | 100.0% | 51.8% | 53.5% | 0.078 |
|  | 5.50 | 2 | 0 | 41 | 43 | 4.7% | 100.0% | 100.0% | 51.2% | 52.3% | 0.152 |
|  | 5.23 | 1 | 0 | 42 | 43 | 2.3% | 100.0% | 100.0% | 50.6% | 51.2% | 0.315 |
| AHI (Supine) | 12.25 | 42 | 42 | 1 | 1 | 97.7% | 2.3% | 50.0% | 50.0% | 50.0% | 1 |
|  | 11.91 | 42 | 41 | 1 | 2 | 97.7% | 4.7% | 50.6% | 66.7% | 51.2% | 0.557 |
|  | 11.66 | 42 | 40 | 1 | 3 | 97.7% | 7.0% | 51.2% | 75.0% | 52.3% | 0.306 |
|  | 11.44 | 42 | 39 | 1 | 4 | 97.7% | 9.3% | 51.9% | 80.0% | 53.5% | 0.167 |
|  | 11.02 | 41 | 39 | 2 | 4 | 95.3% | 9.3% | 51.3% | 66.7% | 52.3% | 0.397 |
|  | 10.67 | 41 | 38 | 2 | 5 | 95.3% | 11.6% | 51.9% | 71.4% | 53.5% | 0.237 |
|  | 10.40 | 41 | 37 | 2 | 6 | 95.3% | 14.0% | 52.6% | 75.0% | 54.7% | 0.138 |
|  | 10.23 | 41 | 36 | 2 | 7 | 95.3% | 16.3% | 53.2% | 77.8% | 55.8% | 0.078 |
|  | 10.12 | 40 | 36 | 3 | 7 | 93.0% | 16.3% | 52.6% | 70.0% | 54.7% | 0.178 |
|  | 10.01 | 40 | 35 | 3 | 8 | 93.0% | 18.6% | 53.3% | 72.7% | 55.8% | 0.107 |
|  | 9.86 | 39 | 35 | 4 | 8 | 90.7% | 18.6% | 52.7% | 66.7% | 54.7% | 0.213 |
|  | 9.71 | 39 | 34 | 4 | 9 | 90.7% | 20.9% | 53.4% | 69.2% | 55.8% | 0.132 |
|  | 9.67 | 38 | 34 | 5 | 9 | 88.4% | 20.9% | 52.8% | 64.3% | 54.7% | 0.243 |
|  | 9.60 | 37 | 34 | 6 | 9 | 86.0% | 20.9% | 52.1% | 60.0% | 53.5% | 0.394 |
|  | 9.54 | 37 | 33 | 6 | 10 | 86.0% | 23.3% | 52.9% | 62.5% | 54.7% | 0.268 |
|  | 9.50 | 36 | 33 | 7 | 10 | 83.7% | 23.3% | 52.2% | 58.8% | 53.5% | 0.417 |
|  | 9.45 | 35 | 33 | 8 | 10 | 81.4% | 23.3% | 51.5% | 55.6% | 52.3% | 0.596 |
|  | 9.32 | 35 | 32 | 8 | 11 | 81.4% | 25.6% | 52.2% | 57.9% | 53.5% | 0.436 |
|  | 9.16 | 35 | 31 | 8 | 12 | 81.4% | 27.9% | 53.0% | 60.0% | 54.7% | 0.307 |
|  | 9.08 | 35 | 29 | 8 | 14 | 81.4% | 32.6% | 54.7% | 63.6% | 57.0% | 0.138 |
|  | 9.05 | 35 | 28 | 8 | 15 | 81.4% | 34.9% | 55.6% | 65.2% | 58.1% | 0.088 |
|  | 9.01 | 34 | 28 | 9 | 15 | 79.1% | 34.9% | 54.8% | 62.5% | 57.0% | 0.149 |
|  | 8.96 | 33 | 28 | 10 | 15 | 76.7% | 34.9% | 54.1% | 60.0% | 55.8% | 0.235 |
|  | 8.90 | 32 | 28 | 11 | 15 | 74.4% | 34.9% | 53.3% | 57.7% | 54.7% | 0.348 |
|  | 8.76 | 32 | 27 | 11 | 16 | 74.4% | 37.2% | 54.2% | 59.3% | 55.8% | 0.245 |
|  | 8.66 | 30 | 27 | 13 | 16 | 69.8% | 37.2% | 52.6% | 55.2% | 53.5% | 0.494 |
|  | 8.62 | 30 | 26 | 13 | 17 | 69.8% | 39.5% | 53.6% | 56.7% | 54.7% | 0.366 |
|  | 8.58 | 29 | 26 | 14 | 17 | 67.4% | 39.5% | 52.7% | 54.8% | 53.5% | 0.501 |
|  | 8.57 | 28 | 25 | 15 | 18 | 65.1% | 41.9% | 52.8% | 54.5% | 53.5% | 0.506 |
|  | 8.55 | 27 | 25 | 16 | 18 | 62.8% | 41.9% | 51.9% | 52.9% | 52.3% | 0.659 |
|  | 8.51 | 27 | 24 | 16 | 19 | 62.8% | 44.2% | 52.9% | 54.3% | 53.5% | 0.510 |
|  | 8.49 | 27 | 23 | 16 | 20 | 62.8% | 46.5% | 54.0% | 55.6% | 54.7% | 0.382 |
|  | 8.47 | 26 | 23 | 17 | 20 | 60.5% | 46.5% | 53.1% | 54.1% | 53.5% | 0.514 |
|  | 8.43 | 26 | 21 | 17 | 22 | 60.5% | 51.2% | 55.3% | 56.4% | 55.8% | 0.279 |
|  | 8.41 | 25 | 21 | 18 | 22 | 58.1% | 51.2% | 54.3% | 55.0% | 54.7% | 0.387 |
|  | 8.40 | 25 | 19 | 18 | 24 | 58.1% | 55.8% | 56.8% | 57.1% | 57.0% | 0.196 |
|  | 8.38 | 24 | 18 | 19 | 25 | 55.8% | 58.1% | 57.1% | 56.8% | 57.0% | 0.196 |
|  | 8.36 | 24 | 17 | 19 | 26 | 55.8% | 60.5% | 58.5% | 57.8% | 58.1% | 0.131 |
|  | 8.34 | 23 | 16 | 20 | 27 | 53.5% | 62.8% | 59.0% | 57.4% | 58.1% | 0.130 |
|  | 8.30 | 23 | 15 | 20 | 28 | 53.5% | 65.1% | 60.5% | 58.3% | 59.3% | 0.082 |
|  | 8.21 | 22 | 15 | 21 | 28 | 51.2% | 65.1% | 59.5% | 57.1% | 58.1% | 0.127 |
|  | 8.09 | 21 | 15 | 22 | 28 | 48.8% | 65.1% | 58.3% | 56.0% | 57.0% | 0.190 |
|  | 7.96 | 20 | 15 | 23 | 28 | 46.5% | 65.1% | 57.1% | 54.9% | 55.8% | 0.272 |
|  | 7.88 | 19 | 15 | 24 | 28 | 44.2% | 65.1% | 55.9% | 53.8% | 54.7% | 0.378 |
|  | 7.86 | 19 | 14 | 24 | 29 | 44.2% | 67.4% | 57.6% | 54.7% | 55.8% | 0.268 |
|  | 7.83 | 19 | 13 | 24 | 30 | 44.2% | 69.8% | 59.4% | 55.6% | 57.0% | 0.181 |
|  | 7.79 | 19 | 12 | 24 | 31 | 44.2% | 72.1% | 61.3% | 56.4% | 58.1% | 0.116 |
|  | 7.69 | 19 | 11 | 24 | 32 | 44.2% | 74.4% | 63.3% | 57.1% | 59.3% | 0.070 |
|  | 7.60 | 19 | 10 | 24 | 33 | 44.2% | 76.7% | 65.5% | 57.9% | 60.5% | 0.040 |
|  | 7.54 | 19 | 9 | 24 | 34 | 44.2% | 79.1% | 67.9% | 58.6% | 61.6% | 0.021 |
|  | 7.49 | 19 | 8 | 24 | 35 | 44.2% | 81.4% | 70.4% | 59.3% | 62.8% | 0.011 |
|  | 7.45 | 18 | 7 | 25 | 36 | 41.9% | 83.7% | 72.0% | 59.0% | 62.8% | 0.009 |
|  | 7.42 | 18 | 6 | 25 | 37 | 41.9% | 86.0% | 75.0% | 59.7% | 64.0% | 0.004 |
|  | 7.36 | 17 | 6 | 26 | 37 | 39.5% | 86.0% | 73.9% | 58.7% | 62.8% | 0.007 |
|  | 7.28 | 16 | 6 | 27 | 37 | 37.2% | 86.0% | 72.7% | 57.8% | 61.6% | 0.014 |
|  | 7.14 | 16 | 5 | 27 | 38 | 37.2% | 88.4% | 76.2% | 58.5% | 62.8% | 0.006 |
|  | 6.95 | 16 | 4 | 27 | 39 | 37.2% | 90.7% | 80.0% | 59.1% | 64.0% | 0.002 |
|  | 6.87 | 16 | 3 | 27 | 40 | 37.2% | 93.0% | 84.2% | 59.7% | 65.1% | 0.001 |
|  | 6.84 | 16 | 1 | 27 | 42 | 37.2% | 97.7% | 94.1% | 60.9% | 67.4% | <0.001 |
|  | 6.81 | 15 | 1 | 28 | 42 | 34.9% | 97.7% | 93.8% | 60.0% | 66.3% | <0.001 |
|  | 6.72 | 14 | 1 | 29 | 42 | 32.6% | 97.7% | 93.3% | 59.2% | 65.1% | <0.001 |
|  | 6.56 | 14 | 0 | 29 | 43 | 32.6% | 100.0% | 100.0% | 59.7% | 66.3% | <0.001 |
|  | 6.44 | 13 | 0 | 30 | 43 | 30.2% | 100.0% | 100.0% | 58.9% | 65.1% | <0.001 |
|  | 6.30 | 12 | 0 | 31 | 43 | 27.9% | 100.0% | 100.0% | 58.1% | 64.0% | <0.001 |
|  | 5.92 | 11 | 0 | 32 | 43 | 25.6% | 100.0% | 100.0% | 57.3% | 62.8% | <0.001 |
|  | 5.60 | 8 | 0 | 35 | 43 | 18.6% | 100.0% | 100.0% | 55.1% | 59.3% | 0.003 |
|  | 5.51 | 7 | 0 | 36 | 43 | 16.3% | 100.0% | 100.0% | 54.4% | 58.1% | 0.006 |
|  | 5.34 | 6 | 0 | 37 | 43 | 14.0% | 100.0% | 100.0% | 53.8% | 57.0% | 0.011 |
|  | 5.22 | 5 | 0 | 38 | 43 | 11.6% | 100.0% | 100.0% | 53.1% | 55.8% | 0.021 |
|  | 4.77 | 4 | 0 | 39 | 43 | 9.3% | 100.0% | 100.0% | 52.4% | 54.7% | 0.041 |
|  | 4.33 | 3 | 0 | 40 | 43 | 7.0% | 100.0% | 100.0% | 51.8% | 53.5% | 0.078 |
|  | 4.03 | 2 | 0 | 41 | 43 | 4.7% | 100.0% | 100.0% | 51.2% | 52.3% | 0.152 |
|  | 3.70 | 1 | 0 | 42 | 43 | 2.3% | 100.0% | 100.0% | 50.6% | 51.2% | 0.315 |

**Appendix 1**: Cut off of the AHI in diagnosing full thickness rotator cuff tears
